# Flow Cytometry: A Blessing and a Curse

**DOI:** 10.3390/biomedicines9111613

**Published:** 2021-11-04

**Authors:** Hannah Drescher, Sabine Weiskirchen, Ralf Weiskirchen

**Affiliations:** 1 Division of Gastroenterology, Massachusetts General Hospital and Harvard Medical School, Boston, MA 02114, USA; 2 Institute of Molecular Pathobiochemistry, Experimental Gene Therapy and Clinical Chemistry (IFMPEGKC), RWTH University Hospital Aachen, D-52074 Aachen, Germany; sweiskirchen@ukaachen.de (S.W.); rweiskirchen@ukaachen.de (R.W.)

**Keywords:** flow cytometry, multiparameter analysis, immunophenotyping

## Abstract

Flow cytometry is a laser-based technology generating a scattered and a fluorescent light signal that enables rapid analysis of the size and granularity of a particle or single cell. In addition, it offers the opportunity to phenotypically characterize and collect the cell with the use of a variety of fluorescent reagents. These reagents include but are not limited to fluorochrome-conjugated antibodies, fluorescent expressing protein-, viability-, and DNA-binding dyes. Major developments in reagents, electronics, and software within the last 30 years have greatly expanded the ability to combine up to 50 antibodies in one single tube. However, these advances also harbor technical risks and interpretation issues in the identification of certain cell populations which will be summarized in this viewpoint article. It will further provide an overview of different potential applications of flow cytometry in research and its possibilities to be used in the clinic.

## 1. Introduction

Flow cytometry first invented in the late 1960s is an extremely versatile and powerful tool for the quantitative and qualitative analysis of cells on a single-cell level [1]. While cells remain in a buffered solution, they flow in a constant fluid stream and pass multiple lasers. Each passing particle is counted and analyzed for the visible light scatter and for multiple fluorescent parameters. The visible light scatter gives information in all directions, but in a typical cytometer, two signals are generated: one using scatter angles near the laser beam direction as a Forward Light Scatter and another using a wide range of angles around 90 degrees. The forward scatter (FSC) indicates the size of a particle, while the side scatter (SSC) displays the granularity of the cell. In addition, individual samples can either be measured based on their expression of fluorescent proteins or can be labeled with dual fluorescent dyes and antibody combinations based on the availability of fluorochromes and dyes [2].

Dyes and machines have undergone tremendous development in recent years leading to impressive growth in the potential number of parameters, which can be used in flow cytometry experiments and analysis. The dramatic evolution in new fluorochromes, fluorescent proteins, tandem dyes, and polymer dyes makes it possible to work with up to 30–50 parameters at once. In addition, advances in machine technology enable the daily use of multiple laser systems not only to analyze cells but also to be used in systems designed for single-cell sorting, systems combining flow cytometry and microscopy, and those that combine flow cytometry and mass spectrometry. In regard to flow cytometry, the protocols can be divided into different classes, namely the isolation of defined cell type or subpopulations, high throughput screening applications, cell surface display, and droplet fluorescent flow cytometry [3].

These technologies have wide applications in molecular biology, pathology, immunology, virology, and many other disciplines. In particular, life cell sorting is extremely valuable and has broad application in medicine including transplantation and reproductive medicine.

The named advances open countless possible applications in various disciplines such as immunology, infectious disease monitoring, molecular or cancer cell biology. Users have the possibility to simultaneously characterize and phenotype cells from different tissues for their protein expression of certain markers and at the same time collect them in bulk or on a single-cell level. With these cells, they have the chance to investigate their mRNA expression profile which creates great opportunities to investigate cell-cell interactions and signaling in different disease settings.

With the increasing number of measured parameters, the way of how analyses are performed had to evolve as well. As the final and one of the most challenging steps, data analysis is traditionally performed by using a two-parameter gating strategy in which the signal of two channels is depicted as a dot plot. Newer ways to analyze complex and high-dimensional data use more sophisticated algorithms that extract useful information [4,5,6,7].

This viewpoint article will provide a brief summary of applications of flow cytometry in research and clinic and present challenges that arise with the fast development of this technique.

## 2. Application in Research and Clinic

Analytical flow cytometry and fluorescence-activated cell sorting (FACS) are closely related procedures in which cell sorting goes a step beyond analytical flow cytometry to obtain samples of bulk or even single cells with analytically defined properties providing highly specific information about individual cells. Analytical flow cytometry can be used for the quantitation of cells and measurement of cellular characteristics, which can for example include cell size, cell cycle status, the expression of specific intracellular markers or receptors, or the identification of protein post-translational modifications. Analytical flow cytometry can be an endpoint in itself. Analytical flow cytometers are usually not all able to perform cell sorting. However, typical cell sorters are all analytical flow cytometers that enable and even require the same measurements and analytic steps to define the cell populations of interest to be sorted by FACS.

The sorting and recovery of cells for post-experimental use requires, besides characteristic fluidics and fluorescence components of a flow cytometer, additional features to divert the cells of interest from within a heterogeneous sample into a separate tube that can be further studied or cultured. Hence, the most important difference and the power of cell sorting compared to analytic flow cytometry is that it opens up a wide variety of potential follow-up investigations using selected cell populations that have known, well pre-defined properties by the initial analytic flow cytometry used to specify the sorting criteria [8,9].

**Figure 1:**
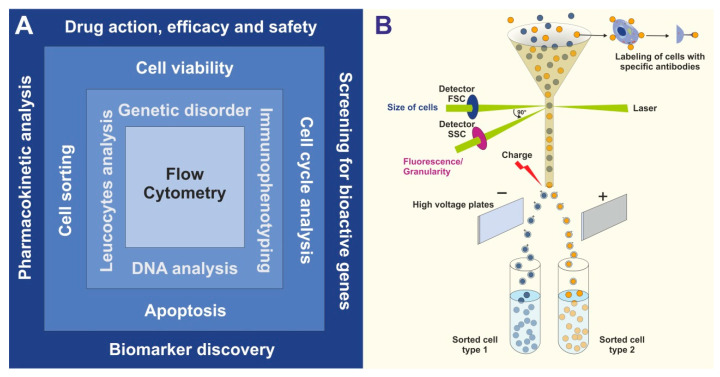
Fields of application and principle of FACS analysis. (**A**) In basic research and clinical routine, FACS is used for many applications including general cell sorting, cell cycle analysis, cell viability testing, apoptosis testing, immunophenotyping, DNA analysis, blood cell analysis, and determination of genetic disorders. (**B**) The cell solution is passed through a narrow channel and cells are subjected to light pulses detected by the forward scattered (FSC) and side scattered (SSC) detectors, which are generated by the transit of the cell through a constant laser source in the flow channel. Besides the characteristics of cells that can already be discriminated by their FSC and SSC profiles, the additional staining with fluorescently-labeled antibodies provides more detailed and specific discrimination between different cell types of interest.

Both analytical flow cytometry and FACS offer multiple applications for research but also in the context of clinical patient evaluation and personalized medicine. The variety of fields to study within different disciplines is broad. There is a variety of conceivable areas of applications of cytometry and FACS (Figure 1A). These range from the identification of genetic disorders, immunophenotyping, DNA analysis, and direct cell analysis of individual cells (e.g., leucocytes). In addition, these methods are the basis for many functional assays including cell viability testing, cell cycle analysis, proliferation assays, apoptosis testing, or cell sorting for more specialized single-cell analysis or transplantation experiments. As such, these technologies have become indispensable for biomedical research and have revolutionized the fields of pharmacology, drug research, gene analysis, and biomarker research. As such flow cytometric experiments can be grouped into a hierarchical order in which (i) either one or a small number of biomolecules can be analyzed such as protein(s) or DNA, (ii) cellular characteristics or individual cells are characterized or phenotyped, or (iii) responses of cells on specific drugs or triggers are defined. Of course, the different levels of this hierarchy are not strictly separated from each other, and boundaries are often blurred.

In particular, the ability to effectively separate and quantify individual cell populations from others by simple and clearly arranged FACS protocols (Figure 1B) is in many cases the starting point for studies investigating specific biological aspects of a defined cell type [10].

The technical advances in the development of flow cytometers have allowed the establishment of a wide spectrum of clinical applications. In primary immunodeficiency diseases (PID), flow cytometry has emerged as an indispensable tool for the evaluation of the immune system and enumeration and characterization of immune cells. In the past, multiple cytometric assays were developed that are now used for diagnostic purposes [11]. Similarly, flow cytometry cannot be omitted in the diagnosis of malignancies developing from lymphocyte precursor cells that often have the same disease spectrum but require a different therapy. Flow cytometry immunophenotyping of peripheral blood cells can detect abnormal cells whose pattern of markers is characteristic for specific types of leukemia and lymphoma [12].

Another example is the dihydrorhodamine (DHR) test introduced in 1988 [13], which provides information about the respiratory burst of the neutrophils after stimulation with phorbol myristate acetate (PMA). In this assay, DHR is oxidated after an appropriate challenge to the green fluorescent compound rhodamine, which can be measured by flow cytometry [13]. As such this assay allows identifying hereditary diseases such as chronic granulomatous disease in which the immune system has difficulties in forming reactive oxygen compounds that are physiologically required to attack certain ingested pathogens [14]. Similarly, the measurement of cell-permeable dyes in cytometric assays allows the analysis of cell cycle and cell proliferation [15,16].

Phenotyping of cells is the most common use of flow cytometry. This technique provides the unique possibility to analyze mixed populations of cells for various markers at the same time. In addition to immune cells from peripheral blood, almost any cell type of any tissue can be isolated, collected, and analyzed in a single cell suspension. Especially for immune cells, phenotyping is regularly performed by staining the cells with specific fluorochrome-conjugates antibodies targeting antigens of interest on the cell surface. Most of these antigens are cluster of differentiation (CD) markers or lineage markers that define certain cell populations. Besides lineage markers, the stained panel can also include different functional markers, which often include intracellular molecules such as transcription factors, cytokines, or proliferation markers that help to further define the immune phenotype and activation state of the cell. Flow cytometers are capable of detecting up to 30 colors in one single experiment. However, the commonly used number of colors that are used presently can be compensated fast and relatively easy range between 15 and 20 color panels for regular immunophenotyping. An example of a Th17 cell immunophenotyping panel consisting out of 23 individual markers is shown in Table 1.

Such immunophenotyping panels can be useful as a diagnostic test for a variety of diseases ranging from early occurring inherited immunodeficiencies to late-stage leukemia [17]. Another simpler but widely applied method for sorting blood cells was established in 1996 by the International Society of Hematotherapy and Graft Engineering (ISHAGE). This protocol is used clinically to quantify the number of peripheral blood stem cells in peripheral blood and apheresis products by staining cells for CD34 representing a transmembrane phosphoglycoprotein expressed on early hematopoietic cells and vascular-associated tissue [18].

In addition to regular immunophenotyping, antigen-specific responses of immune cells can be measured via flow cytometry as well. For these experiments, cells are stimulated with a specific antigen and analyzed for their activation detected by cytokine expression, expression of different memory markers, or their antigen recognition through major histocompatibility complex (MHC) class I and II multimers. These multimers consist of MHC-I or MHC-II monomers, which are assembled to either tetramers, pentamers, or dextramers bound to a fluorescent backbone. Multimers can be loaded with any antigen of choice and are used to detect antigen-specific T cells. This technique is widely used in vaccine studies and makes it even possible to detect extremely rare T cell populations such as hepatitis B virus-specific T cells in chronic patients.

Other commonly used measures with flow cytometry are proliferation, cell cycle, and apoptosis assays. Cell proliferation can be detected using either cell-permeable dyes that are inheritable such as carboxyfluorescein succinimidyl ester (CFSE), thymidine analogs that incorporate into replicating DNA such as bromodeoxyuridine (BrdU), or with the use of proliferation-associated antigen 67 (Ki-67) expression.

**Figure 2:**
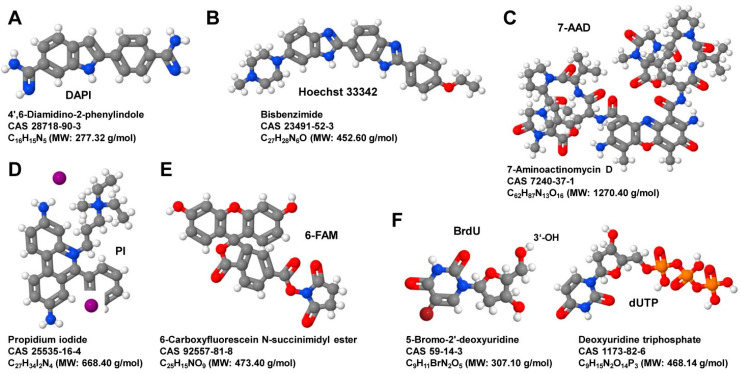
Selection of substances frequently used in FACS analysis. (**A**) 4′,6-dDiamidino-2-phenylindole (DAPI) is a cell-permeable compound that binds strongly to adenine–thymine-rich regions in DNA. The DAPI-DNA complex has an absorption maximum at a wavelength of 358 nm (ultraviolet) and an emission maximum at 461 nm (blue) that can be detected through a blue/cyan filter. (**B**) Unbound Hoechst 33,342 has a maximum fluorescence emission at 510–540 nm, while this dye emits in ultraviolet light (~350 nm) blue fluorescence with an emission spectrum maximum at 461 nm when bound to double-stranded DNA. (**C**) The fluorescent dye 7-Aminoactinomycin D intercalates primarily in double-stranded DNA with high GC-content. If used for cell staining in FACS, the cell membrane must be permeabilized. It can be best excited with diode lasers at 561 nm resulting in an emission with a maximum of 647 nm. (**D**) Propidium iodide is a not membrane-permeable fluorescent DNA intercalating agent with an excitations maximum of 493 nmg (blue-green), and an emission maximum of 636 nm (red). (**E**) 6-Carboxyfluorescein *N*-succinimidyl ester is a cell-permeable, amine-reactive fluorescent dye that particularly binds via its succinimidyl group to proteins rich in lysine with an absorption wavelength of 495 nm and an emission wavelength of 517 nm. (**F**) 5-Bromo-2′-deoxyuridine (left) is a nucleotide analog to dUTP (right) that can incorporate in place of thymidine in newly synthesized DNA. The modified DNA can be detected with BrdU-specific antibodies or directly visualized when labeled with flurochormes at its 3′-OH position. All images were generated with the open-source Java viewer Jmol version 14.2.15_2015.07.09 [19].

Cell cycle analyses are performed by measuring DNA with a DNA binding dye. Most common are propidium iodide (PI) and 4′,6′-diamidino-2-phenylindole (DAPI) and 7-amino-actinomycin D (7-AAD) (Figure 2). Cell apoptosis is measured with assays that target the activation of caspases, mitochondrial apoptosis and chromatin condensation (Hoechst 33342), endonuclease digestion of DNA (i.e., terminal deoxynucleotidyl transferase dUTP nick end labeling, TUNEL), or the translocation of the plasma membrane (Annexin V).

Currently, there is a great abundance of suitable dyes for cytometric analysis. The different organic dyes, fluorescent proteins, and tandem conjugates differ in excitation and emission maxima and can be used in many applications (Table 2). Some of them can freely penetrate the cell membrane, while others cannot penetrate live cell membranes. In addition, the dyes differ in their stability towards light, pH, and fixatives, requiring special attention to experimental planning.

Highly innovative flow cytometry-based single-cell analysis and single-cell sorting approaches slowly start to transition from being solely used in basic research projects. Actually, there are many efforts to implement protocols into clinical applications. So, likewise, numerous other molecularly techniques, flow cytometry and, in particular, FACS, is a significant add-on for stratifying patients to personalized treatments. In particular, many cancer-based clinical studies have shown that these methodologies offer novel avenues for precision personalized medicine for the treatment of cancer patients [20].

The ability to characterize cell types under normal homeostatic conditions but also in different disease stages makes it possible to detect cell types and signaling pathways involved in disease development and progression. It might even show whether a patient will respond to a certain therapy and/or is at risk to develop severe complications upfront. Flow cytometry and downstream application will thus be an important technique driving future therapeutic developments.

In addition to clinical advances, these methodologies have brought, corresponding methods are nowadays indispensable for daily research. They offer a number of practical applications starting including the measurement of transfection efficiencies when a fluorescent protein is used as a marker, detection, and quantification of cellular apoptosis, cell proliferation, cell cycle analysis, live/death cell discrimination, immunophenotyping of various immune cells, and of course the isolation of specific cell subsets for further analysis.

## 3. Potential Challenges

With the fast development of flow cytometers and reagents also challenges arise during the different experimental and analytical stages including the choice of reagents and antibodies, the preparation and storage of cells to be analyzed, the overall experimental plan, and of course the final data analysis [21]. Flow cytometry bears a lot of execution and interpretation risks starting with preanalytical and experimental issues through analytical difficulties as far as interpretation concerns. In this section, we want to point out potential challenges which are commonly seen in samples processing and data interpretation.

### 3.1. Experimental Issues

Before even starting an experiment, the investigator defines his/her experimental hypothesis to be able to choose and design the best panel to obtain all necessary information [15]. Typical questions to be asked are: (i) Which cell populations should be investigated and how many markers are needed to properly define these populations? (ii) Are the specific markers of interest expressed on the cell surface or intracellular? (iii) Are the stained antigens expressed with high or low density? (iv) What is the proper gating strategy, meaning which area on the scatter plot should be selected to distinguish cells of interest?

The next crucial step is sample collection. Especially when working with tissue samples, processing time and the digestion method are important steps that are imperative for the success of the experiment. Therefore, the researcher must be aware of specific characteristics of the handled tissue and cell type so that the cell isolation method can be optimized to the smallest detail. Especially when working with human tissue, consistency, and optimal sample quality are important as the amount of available tissue is in most cases very low and repeating the experiment is not an option. Sample quality must be critically evaluated to ensure good cell viability and accurate results. Cell clotting and hemolyzation and the media in which cells are stored can cause problems. While staining, samples need to be thoroughly washed to reduce the background noise and should be stained in a buffer containing agents to block unspecific antibody binding. Adding fresh DNAse or ethylenediaminetetraacetic acid (EDTA) to the staining buffer can further prevent cells from clotting. Cell viability is a usual concern when working with tissue samples. Viability staining should be performed for all samples to properly exclude debris and ensure an accurate assessment of sample quality and consistency.

**Figure 3:**
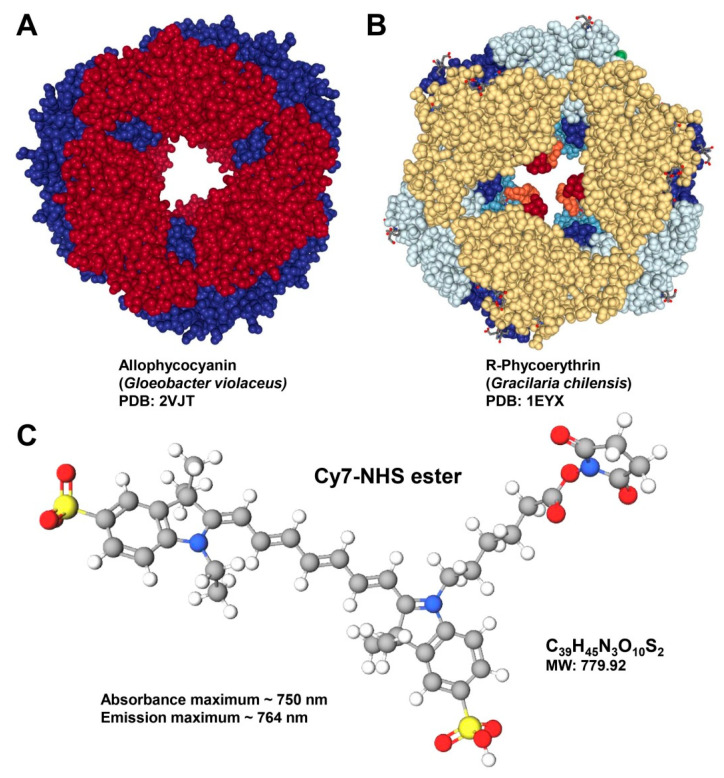
Examples of pigments with fluorescent properties. (**A**) Allophycocyanin (APC) is an accessory, water-soluble pigment of red or blue-green algae that has an absorption maximum at 652 nm and an emission maximum at 657.5 nm. It is composed of a trimer with an (αβ)_3_ structure and a molecular weight of 105,000 Daltons. The structure of the Allophycocyanin from *Gloeobacter violaceus* depicted was taken from the RCSB Protein Data Bank (PDB, access. no.: 2VJT) (**B**) Phycoerythrin (PE) is a red protein-pigment occurring in red algae and cryptophytes. It is composed of αβ monomers that can form either trimers (αβ)_3_ or hexamers (αβ)_6_. Depicted is the crystal structure of the light-harvesting R-Phycoerythrin from the red algae *Gracilaria chilensis* deposited in the PDB (access. no. 1EYX). The structures shown in (**A**,**B**) were created with the NGL WebGl-based molecular viewer [24]. (**C**) Structure of the cyanine 7-*N*-hydroxysuccinimide (Cy7-NHS) ester that is a near-infrared fluorescent dye specifically designed for labeling of amines.

Another issue that can occur is the breakdown of antibody tandem dyes, a condition that affects the variability in emission spectra resulting in a considerable decrease in reliability [22]. Antibody tandem dyes are complexes of two covalently attached fluorescent molecules or fluorophores, in which one serves as the donor and the other as the acceptor. Examples for such tandem-dyes are allophycocyanin conjugate-cyanine 7 (APC-Cy7) or phycoerythrin-cyanine 5.5 (PE-Cy5.5) that are, for example, composed of a large light-harvesting pigment (APC, PE) and a synthetic dye such as a cyanine (Figure 3).

While some of these tandems are very stable, others can dissociate over time. This disintegration results in the false positive detection of the signal in the parent channel which would here be APC or PE. To prevent this degradation, antibodies should always be kept in the dark, at appropriate temperate (4 °C), and used as fresh as possible. In addition, such circumstances demonstrate the need for better standards in flow cytometry and critical control of reagents [15]. Moreover, the quality of tandem reagents might be highly variable from lot to lot, which can lead to significant differences and background noises affecting particularly the accuracy of the signal when more than one fluorochrome is used to stain cells [15]. This phenomenon resulting from the physical overlap among the emission spectra of the different fluorochromes, which is commonly called spectral spillover, has to be corrected electronically [23].

### 3.2. Analytical and Interpretation Pitfalls

**Figure 4:**
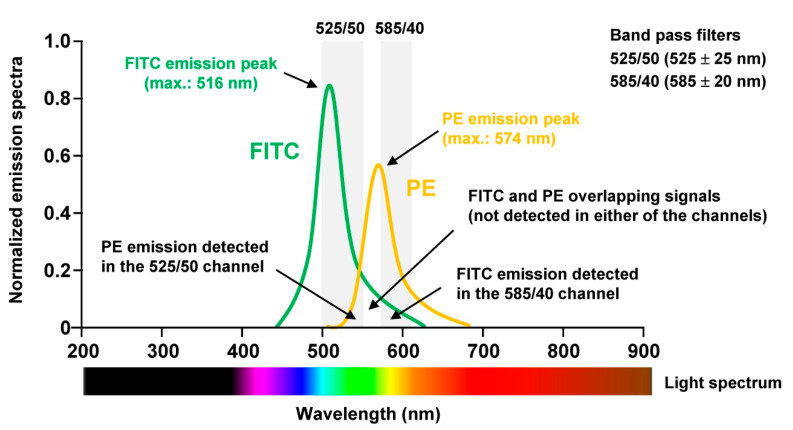
Fluorescence compensation. The fluorescence emission spectra for Fluorescein isothiocyanate (FITC) and Phycoerythrin (PE) partially overlap when detected with the commonly used filters 525/50 (allows to pass wavelengths with an entire band’s width of 525 ± 25 nm) and 585/40 (allows to pass wavelengths with an entire band’s width of 585 ± 20 nm). When FITC or PE should be properly quantified using so-called ‘compensation controls’, which are single stained samples for each used fluorochrome within the respective panel, the signals that result from the other fluorochrome in the respective channel of the band pass filters used for measurement must be subtracted. FITC and PE overlapping signals that are not detected in either of the channels can be ignored [25].

To perform proper analysis especially of human tissue samples not only the panel has to be chosen wisely, the sample quality checked properly, but the machine must be optimally aligned. For optimal signal detection, photomultiplier tubes (PMT) are commonly used, which are extremely sensitive detectors of light in the ultraviolet, visible, and near-infrared range. Moreover, it is necessary to correct fluorescence spillover. This is done in a process termed compensation and refers to the procedure in which signals of any given fluorochrome are removed from all detectors except the one devoted to measuring the respective dye. When using for example the combination of Fluorescein isothiocyanate (FITC) and Phycoerythrin (PE) in one experiment, the relative contribution of each fluorophore to the measured signal in each PMT must be determined because the spectra of the two dyes partially overlap (Figure 4).

Proper setting of the PMT voltages is necessary to maximize signal-to-background resolution. A voltage that is set too low will result in suboptimal photoelectron generation and signal detection, while a voltage that is set too high results in bright signals falling out of the linear range of detection.

Because compensation in FACS analysis is often misunderstood or misapplied, incorrectly compensated data are often the reason for misinterpretation of experimental data or the miscalculation of specific cell subsets in complex solutions.

**Figure 5:**
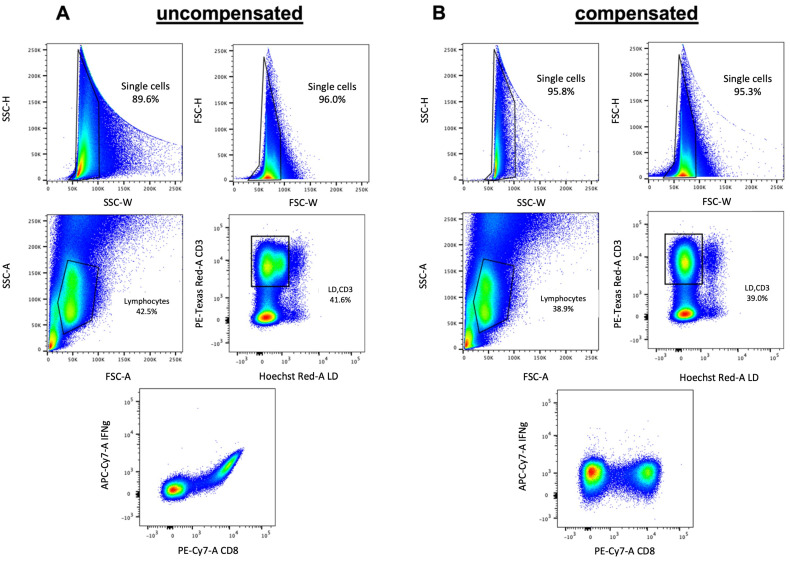
Analytical pitfalls in FACS analysis. When working with a 15–20 color panel, compensation is of utmost importance. (**A**) shows the gating of a human peripheral blood mononuclear cells (PBMCs) sample before compensation. (**B**) displays the same file after automatically applying the compensation in FlowJo, a software application that has an integrated environment for viewing and analyzing flow cytometric data [26]. Due to the nature of flow cytometry, the emission of a specific dye in a mixture of dyes is usually measured not only in a single detector but in all detectors being used in the specific experiment. During compensation, the signals of any other fluorochrome are removed except the one devoted to measuring that dye by calculating and subtracting the spillover values of them. Therefore, for each fluorochrome used in the experiment, a single-stained sample must be analyzed in parallel. Although the need for compensation can be avoided by using fluorophores that do not have overlapping emission spectra or combining fluorophores that can be uniquely activated by specific laser lines, compensation will be usually necessary when using panels with a large number of different fluorochromes.

Fluorescence compensation is especially crucial when working with 15–20 color panels compensation becomes of tremendous importance and can only hardly be done manually (Figure 5). Computer algorithms can perform the compensation quickly and easily with single stained samples for every used color.

After the acquisition of the sample, there are different analytic variables that must be taken into consideration to ensure consistent high-quality data of the flow cytometry experiment. Conventional analysis of flow cytometry data starts with the exclusion of doublets that can severely compromise the accuracy and interpretation of analytical results, for example, by producing a biologically incorrect ‘double positive’ population consisting of doublets, thereby also comprising the purity of a cell sort. The flow cytometer can detect doublets because they show a long time of flight through the machine and have a different pattern in the light scatter. By plotting FSC versus the forward amplitude doublets can easily be excluded from the analysis. After removal of debris and doublets, data analysis usually consists of comparing one or two different measured parameters and setting gates around the cell population of interest. This selects or excludes specific cells of interest that can then be further analyzed for the expression of other markers included in the panel. Typical examples for cells that are excluded from the analysis are dead cells that are positively stained with a dye or groups of cells that are not of interest, which can all be stained in the same color. This allows the exclusion of different cell populations that would interfere with the analysis at a time without blocking more than one color in the panel.

Especially human samples show a lot of inter-sample variability, which makes proper gating difficult sometimes. To make sure that the gate is set at the correct position, even if the population does not show a clear cloud, is the option to acquire a fluorescence minus one (FMO) tube for the panel of choice. This FMO includes all used antibodies but one so that it gets easy to see above which threshold the specific antibody shows a positive signal. Moreover, background levels can be another problem, especially when investigating cells with low expression levels for the used marker. In such cases, extensively validated isotype controls raised against an antigen not found on the cell type of interest are essential to determine the level of background staining and to ensure that the observed signal is due to specific antibody binding to the proper target molecule. In addition, antibodies have a tendency to stick to dead cells, a phenomenon resulting in the impure viability of sorted cells.

## 4. Conclusions

Flow cytometry is a very powerful technology to phenotype and characterizes cells on a single-cell level. Recent advances in technology have made the collection of flow cytometry data a daily routine, but consistent high-quality analysis and interpretation are still challenging. Given the collection of hundreds of thousands of events in minutes, the choice of the measured parameters, the gating strategy, and statistical analysis gives incredible opportunities but also bears certain risks. Flow cytometry data have to be further considered in the appropriate clinical context. Especially human datasets have to be handled with care as in some cases the homeostatic conditions are not well-defined, and normal patterns are not at all static. In addition, antigen expression can easily change during different types of treatments so that parameters such as time after treatment, dosage, and basic patient characteristics need to be critically considered. This makes it extremely challenging to evaluate and judge data in a clinical and/or therapeutic setting.

## Figures and Tables

**Table 1:** Immunophenotyping of human Th17 cells by flow cytometry.

Marker	Protein	Localization	Function
CCR4/CD194	C-C chemokine receptor type 4	Cell surface	Chemokine receptor
CCR6/CD196	C-C chemokine receptor type 6	Cell surface	Chemokine receptor
CD3	cluster of differentiation 3	Cell surface	T cell coreceptor
CD4	cluster of differentiation 4	Cell surface	T cell coreceptor
CD8	cluster of differentiation 8	Cell surface	T cell coreceptor
CD14	cluster of differentiation 14	Cell surface	LPS and PAMP receptor
CD19	cluster of differentiation 19	Cell surface	Adapter protein
Il1R1/CD121a	Interleukin 1 receptor type 1	Cell surface	Cytokine receptor
IL-6Rα/CD126	Interleukin 6 receptor type α	Cell surface	Cytokine receptor
IL-21R/CD360	Interleukin 21 receptor	Cell surface	Cytokine receptor
IL-23R	Interleukin 23 receptor	Cell surface	Cytokine receptor
TGFβRII	Transforming growth factor-β receptor type II	Cell surface	Cytokine receptor
Batf	Basic leucine zipper ATF-like transcription factor	Intracellular	Transcription factor
IRF4	Interferon regulatory factor 4	Intracellular	Transcription factor
RORα/NR1F1	RAR-related orphan receptor α	Intracellular	Nuclear receptor
RORγt/RORC	RAR-related orphan receptor γ	Intracellular	Nuclear receptor
STAT3	Signal transducer and activator of transcription 3	Intracellular	Transcription factor
CCL20/MIP-3α	C-C motif ligand 20	Secreted	Chemokine
IL-17A	Interleukin 17A	Secreted	Cytokine
IL-17F	Interleukin 17 family	Secreted	Cytokine family
IL-21	Interleukin 21	Secreted	Cytokine
IL-22	Interleukin 22	Secreted	Cytokine
IL-26	Interleukin 26	Secreted	Cytokine

**Table 2:** Representative fluorochromes used for flow cytometry.

Fluorochrome	Excitation Maximum	Emission Maximum	Remarks
Pacific Blue	401 nm	455 nm	Recommended for highly expressed antigens
Alexa Fluor 405	401 nm	421 nm	pH insensitive
eFluor 450	405 nm	450 nm	Stable fluorescence also after aldehyde fixation
Alexa Fluor 488	495 nm	519 nm	Bright dye with high photostability, suitable for intracellular staining
FITC	494 nm	520 nm	Sensitive to pH changes and photobleaching, reserved for highly expressed markers
PE	565 nm	578 nm	Poor photostability, excitable at many wavelengths
CFSC	494 nm	521 nm	Cell permeable dye used to track cell proliferation
PI	536 nm	617 nm	Intercalating agent that binds non-specifically to DNA and RNA, penetrates disrupted membranes
PerCP	477 nm	678 nm	Highly susceptible to photodegradation
PE/Cyanine 5	565 nm	667 nm	Tandem conjugate sensitive to photobleaching, exhibit non-specific binding to Fc receptors
PE/Cyanine 7	565 nm	785 nm	Tandem conjugate sensitive to light exposure and paraformaldehyde fixation
APC	650 nm	660 nm	Exhibits far-red fluorescence with high quantum yields
Alexa Fluor 647	650 nm	668 nm	Small molecule dye with good photostability, useful for intracellular applications
Alexa Fluor 660	663 nm	690 nm	Small molecule dye with good photostability
APC/Cyanine 7	650 nm	785 nm	Tandem conjugate, sensitive to light and fixation
APC-Cyanine 5.5	651 nm	680 nm	Tandem conjugate, popular in color used in many flow cytometric applications

Abbreviations used are: APC, allophycocyanin; CFSE, carboxyfluorescein diacetate; FITC, fluorescein isothiocyanate; PE, R-phycoerythrin; PerCP, peridinin chlorophyll; PI, propidium iodide.
